# Exposure to polystyrene nanoparticles leads to changes in the zeta potential of bacterial cells

**DOI:** 10.1038/s41598-023-36603-5

**Published:** 2023-06-12

**Authors:** Marcin Zając, Joanna Kotyńska, Grzegorz Zambrowski, Joanna Breczko, Piotr Deptuła, Mateusz Cieśluk, Monika Zambrzycka, Izabela Święcicka, Robert Bucki, Monika Naumowicz

**Affiliations:** 1 Doctoral School of Exact and Natural Sciences, University of Bialystok, 1K K. Ciolkowski Str., 15-245 Białystok, Poland; 2 Laboratory of Bioelectrochemistry, Department of Physical Chemistry, Faculty of Chemistry, University of Bialystok, 1K K. Ciolkowski Str., 15-245 Białystok, Poland; 3 Laboratory of Molecular Biophysics, Department of Microbiology and Biotechnology, Faculty of Biology, University of Bialystok, 1J K. Ciolkowski Str., 15-245 Białystok, Poland; 4 Laboratory of Applied Microbiology, Department of Microbiology and Biotechnology, Faculty of Biology, University of Bialystok, 1J K. Ciolkowski Str., 15-245 Białystok, Poland; 5 Laboratory of Materials Chemistry, Department of Physical Chemistry, Faculty of Chemistry, University of Bialystok, 1K K. Ciolkowski Str., 15-245 Białystok, Poland; 6 Department of Medical Microbiology and Nanobiomedical Engineering, Medical University of Bialystok, 2C A. Mickiewicz Str., 15-222 Białystok, Poland

**Keywords:** Membrane biophysics, Nanoscale biophysics, Chemistry, Nanoscience and technology

## Abstract

Polymer molecules, the main components of plastics, are an emerging pollutants in various environmental compartments (water, air, soil) that may induce several ecotoxicological effects on live organisms. Therefore, understanding how plastic particles interact with bacterial cell membranes is crucial in analysing their associated risks in ecosystems and human microbiota. However, relatively little is known about the interaction between nanoplastics and bacteria. The present work focuses on *Staphylococcus aureus* and *Klebsiella pneumoniae*, representing the Gram-positive and Gram-negative bacteria respectively, exposed to 100 nm diameter polystyrene nanoparticles (PS NPs). The nanoparticles attach to the cells’ membranes of both bacteria, changing their electrical charge, but without the effect of killing the cells. PS NPs caused a change in zeta potential values (both species of bacterial strains), dependent on particle concentration, pH, as well as on exposure time of bacteria to them. Through the application of AFM and FTIR techniques, the presence of PS NPs on bacterial surfaces was detected, suggesting the affinity of the particles to bacterial components, but without any changes in the morphology of the tested bacteria. The zeta potential can be more widely used in the study of interactions between nanostructures and cells.

## Introduction

Plastics are the most important class of man-made, unnatural products that significantly penetrate the Earth's surface. These durable synthetic organic polymers are a potential indicator for the Anthropocene, an epoch of time in which humans dominated many surface geological processes on the Earth^1^. Plastic waste, defined as plastic objects present in the environment that do not fulfill their intended function, is durable, mobile and ubiquitous on land and water, including cities, rural areas and even natural environments with limited human activity. The large plastic debris is easily visible and adversely affects wildlife by directly entangling, ingesting and injuring a wide variety of animals.

Microplastics are considered a plastic debris in a micro scale with particles size of less than 5000 µm in length. Until recently, these particles have been a largely overlooked part of plastic pollution. However, this attitude has changed in the last decade, during which research focusing on the formation, characteristics, further fragmentation, chemical interactions, environmental fate, and potential impact of microplastics has been increasingly developed^2^. Plastic particles ranging from 1 to 1000 nm refer to as nanoparticles (NPs)^3^. Another definition of NPs includes particles smaller than 100 nm in at least one dimension^4^. In fact, this definition is related to the current definition of manufactured nanomaterials, but it remains inappropriate from the point of view of colloidal physics and chemistry^3^. The NP particles are formed indirectly, through the fragmentation of larger plastic specimens, and directly, from daily-use products, such as cosmetics and some pharmaceuticals. Although the direct observational evidence supporting these hypotheses is lacking^5^, the literature reports of physical transformations leading to the formation of monogranular NP-type particles, such as light-induced degradation^6^ or mechanical disintegration of PS^7^. However, the chemical composition of the resulting particles has not yet been documented.

The physicochemical characterization of nanoplastics is crucial in understanding their behavior in natural systems. With an extremely high surface-to-volume ratio, surface properties dominate the physical, chemical, and biological interactions of nanoplastic and will determine their environmental fate. In the environment, and after their uptake into plants and animals, nanoplastics’ surfaces will interact with surrounding inorganic and organic matter. This will lead to agglomeration and different surface chemistry that will influence the exposure scenarios and secondary interactions with biological systems, and, therefore, produce biological effects^7^. Agglomeration, strongly dependent on temperature, dissolved organic matter, and ions, can alter their transport and fate in the aqueous environment. Homo-and hetero-aggregation can facilitate sedimentation and separation from water bodies, modifying the bioavailability of different aquatic organisms. Since computational modeling is considered auxiliary in predicting fate under various environmental conditions, the transport of nanoparticles in aqueous dispersion has often been modeled using Derjaguin–Landau–Verwey–Overbeek (DLVO) theory. However, due to the complex nature of nanoplastics, the theory may not always accurately describe their behavior. Nanosized particles' surface charge and electrostatic activity usually differ from those in the bulk material^8,9^. Therefore, dynamic light scattering (DLS), which is used to determine the hydrodynamic diameter of the nanoplastics in solution, is a valid method of detecting the agglomeration state^9,10^.

Polystyrene (PS) is a synthetic glassy polymer with a high molecular weight and linear structure, industrially prepared by radical polymerization of styrene. PS is a physiologically safe and low-cost thermoplastic extensively used to produce disposable tableware, medical equipment, or packaging. The large production scale of PS and its biodegradability resistance leads to the contamination of all ecosystems with this polymer. PS degradation involves colonization of its surface by microorganisms, followed by the action of exoenzymes that degrade the polymer to oligomers or even monomers^11^. The attention of a large number of scientists has focused on research on plastics as habitats for biofilm-forming microorganisms. Biofilms protect microorganisms from UV radiation, high salinity, heavy metals, or antibiotics, increasing their microbiological activity^12^. It is worth mentioning that many previous research have studied polystyrene nanometer-sized particles (PS NPs) e.g.^13,14^. Nevertheless, due to their small size, large surface area, chemical composition, and other physicochemical properties, they interact with biological systems via mechanism(s) that still need to be understood^15^.

The cytoplasmic membrane of most bacteria is surrounded and sustained by a cell wall which assures its strength, rigidity, and shape. The bacterial wall is a heterogeneous structure consisting of numerous macromolecules such as lipids, proteins, lipopolysaccharides, and teichoic acid^16^. Nevertheless, an essential component of the wall is peptidoglycan. While, the wall of Gram-positive bacteria consists of a rigid and relatively thick layer of peptidoglycan with adherent teichuronic and teichoic acids^17^, in Gram-negative bacteria the peptidoglycan layer is much thinner but covered with an outer membrane containing lipopolysaccharides^18^. Macromolecules present in the cell wall and bacterial membranes contribute to the surface charge of bacterial cells through the ionization of functional groups, such as carboxyl, phosphate, amino, and hydroxyl groups^19^.

The charge on the bacterial cell surface determines electrostatic interactions between the cell surface and a particle. This parameter, together with the size of the particle, establishes whether the particle attaches to the cell surface or internalizes into the cells^20^. Most bacteria possess a net negative surface charge, compensated by oppositely charged ions in the surrounding environment^21^. Since direct measurement of the surface charge is challenging, the zeta potential (also known as electrokinetic potential), given as the *ζ* value, is an important indicator of understanding these dependencies properly.

The zeta potential is the electrical potential difference at the hydrodynamic slipping surface, described as the interface between the aqueous liquid and the stationary layer of fluid adjacent to the bacterial cell surface, and determined from electrophoretic mobility measurements^22^. The zeta potential plays a substantial role in the preservation of cellular functions, and also provides valuable information about cell surface characteristics^23^. In some cases, the interaction between the bacterial surface and various factors can be regulated by electrostatic interactions that in turn can influence zeta potential, which subsequently alter the permeability of the cell surface leading to the cell death.

It was hypothesized that polystyrene nanoparticles (PS NPs) attach to bacteria altering their surface properties, similar to negatively charged titanium dioxide nanoparticles^24^ or neutral polyethylene oxide particles^25^. Thus, the objective of the work described here was to verify whether the interaction between 100 nm PS NPs and bacteria could potentially affect the surface potential (represented as zeta potential) of bacteria. The bacteria used in this study, purchased from the American Type Culture Collection (ATCC), included Gram-positive *Staphylococcus aureus* strain ATCC 6538 and Gram-negative *Klebsiella pneumoniae* strain ATCC 4352. The antimicrobial efficacy of nanoparticles against these bacteria was evaluated by determining both minimum inhibitory concentration (MIC) and minimum bactericidal concentration (MBC). The bacterial zeta potential, pH, polystyrene concentration, the exposure time of the bacteria to the nanoparticles, and ionic strength of the suspending medium were varied to analyze different aspects of the cell-polymer interactions. In addition, atomic force microscopy (AFM) was employed to record the topography and height images of bacteria treated with nanoparticles. Furthermore, the interaction between bacteria and PS NPs was assessed using Fourier transform infrared (FTIR) spectroscopy. To our knowledge, hardly any sources provided in the subject’s literature undertake such a broad experimental approach. Okshevsky et al.^26^ analysed the impact of nanoparticles’ surface characteristics and their concentration on biofilm formation by marine bacteria. The authors investigated two types of nanoparticles’ suspensions and seven different bacteria. Ly et al.^27^, however, examined the impact of three *Lactic acid* bacterial strains on the stability of model emulsions. Using the MATH method and microelectrophoresis, they characterized hydrophobic and electrostatic cell-surface properties.

## Materials and methods

### Materials

The polystyrene nanoparticles were purchased from Sigma Aldrich (Saint Louis, United States), catalog no. 43302. According to the manufacturer, the PS NPs size was 100 nm (standard deviation ≤ 0.01 μm), and the density was 1.05 g/ml. The PS NPs were packaged as 10% solids aqueous suspensions.

Chemicals used in the investigation, e.g., glycerol, sodium chloride, potassium bromide, sodium hydroxide, poly-l-lysine, and hydrochloric acid were purchased from Sigma Aldrich (Saint Louis, United States), while ethanol from Merck (Darmstadt, Germany). All chemicals had an analytical purity and were used without further purification.

Microbiological media, e.g., Mueller–Hinton broth (MHB), Mueller–Hinton agar (MHA), Luria–Bertani (LB) broth, nutrient agar, and brain heart infusion (BHI) agar, were obtained from Oxoid Ltd. (Basingstoke, UK).

Deionized water (R = 18 MΩ cm^−1^) used for electrode rinsing was obtained from a Milli-Q water purification system (Millipore, Bedford, MA, USA).

### Experimental

#### Polystyrene nanoparticles characterization

The particle diameter, dispersity, and zeta potential were characterized by AFM, dynamic light scattering (DLS) and electrophoretic light scattering (ELS) techniques using the Zetasizer Nano-ZS instrument (Malvern Instruments Ltd, Malvern, United Kingdom). Accordingly, PS NPs were diluted in distilled water or 0.3, 30, 100, and 155 mM NaCl to a final concentration of 20 μg/ml. Subsequently, suspensions were homogenized by sonication immediately before conducting further tests using a probe sonicator for 20 min (Techpan, Warsaw, Poland). Measurements were conducted at pH 7.4 and 25 °C. The pH was adjusted to the required value using NaOH.

#### Bacteria preparation process

The microorganisms utilized in this study, *S. aureus* strain ATCC 6538 and *K. pneumoniae* strain ATCC 4352, kept at − 80 °C in LB broth and glycerol (1:1), were inoculated on a nutrient agar plates, incubated at 37 °C overnight, and stored at 4 °C for further usage. A single isolated colony was picked from the plate, transferred to MHB, and incubated at 37 °C for 24 h. The bacterial cell suspension was prepared by resuspending the cell pellet in a background solution (distilled and autoclaved water or NaCl solution at the same ionic strength as in the respective experiment). The bacterial suspensions were centrifuged at 8,000 rpm (20 min), the supernatant was discarded, and the cell pellets were washed five times with background solution to ensure complete removal of the growth medium particles (unless otherwise specified—“Minimum inhibitory and minimum bactericidal concentrations for bacteria treated with polystyrene nanoparticles” section). The OD_600_ of the final dispersion, measured with a V-670 spectrophotometer (Jasco Corp., Tokyo, Japan), varied between 0.10 and 0.12.

#### Determination of the minimum inhibitory concentration (MIC) and the minimum bactericidal concentration (MBC)

The MIC values were determined in accordance with the Clinical and Laboratory Standard Institute (CLSI) protocol^28^. Briefly, the PS NPs solution at a concentration of 1.05 g/ml was dispersed in MHB in a U-shaped 96-well microtiter plate within a concentration range 2.39∙10^–13^–1.05∙10^3^ g/ml (arithmetic scale). The bacteria were cultured overnight in MHB at 37 °C with shaking at 160 rpm and then suspended to a final optical density of 0.2–0.3 at 600 nm wavelength measured with a V-670 spectrophotometer (Jasco Corp.). Then, 100 μl of the bacterial suspensions was added to each well in the microtiter plate containing diluted PS NPs. Bacteria in plates were incubated overnight at 37 °C. The MIC values were determined as the lowest concentration of the PS NPs in the wells with no bacterial growth observed visually. All the tests were carried out in quadruplicate, and the results were averaged.

In order to assess the MBC values of the PS NPs extracts, 5 μl of the overnight culture from each well in the microtiter plate with the PS NPs of a concentration equal to and higher than the MIC value, were inoculated onto BHI agar with the use of a sterile plastic spreader, and incubated overnight at 37 °C. The MBC values were determined as the lowest concentration of the extracts in the wells with no bacterial growth on the plates observed visually. All the tests were carried out in quadruplicate, and the results were averaged. In the experiments, bacteria cultured in BHI without the PS NPs, were considered as positive control.

#### Zeta potential measurements

The zeta potential of the intact cells and those subjected to treatment with PS NPs was measured as a function of pH by the ELS technique using a Zetasizer Nano ZS analyzer (Malvern Instruments Ltd, Malvern, United Kingdom). As such, 0.2 ml of washed bacterial suspension was dispersed in 0.8 ml of the desired concentration of NaCl. The pH was adjusted to the required value (range 3–11) using HCl or NaOH with the same ionic concentration as for the cell suspension. The suspension was stirred and conditioned until the pH stabilized. The resulting suspension was then used to fill a transparent, disposable folded capillary zeta cell coupled with gold-plated electrodes (Malvern Instruments Ltd), and the measurement was proceeded. Between successive measurements, the electrodes were rinsed with copious amounts of ethanol and deionized water, followed by the tested bacteria suspension. Six *ζ* measurements were made (each covering 100–200 runs with a duration of 2 s) at the given pH value and the temperature of 25 °C. The experiments were repeated at least three times, and the average values are reported here.

Various concentrations (0.3, 30, 100, and 155 mM) of NaCl were used to study the effect of different ionic strengths on the *ζ* values of bacterial cells. To examine the effect of polystyrene particles treatment on *S. aureus* and *K. pneumoniae*, PS NPs were added to 0.3 mM NaCl, sonicated for 20 min, and then mixed with the bacterial cell suspension to achieve final polystyrene concentrations of 0.4, 2, 20, and 100 μg/ml. The mixture was incubated (25 °C) in a shaking bath for further 20 min before the *ζ* measurements.

In order to study the influence of bacterial exposure time to PS NPs on the ***ζ*** of their cells, the suspension of bacteria and PS particles at selected concentrations prepared in 0.3 mM NaCl was applied. Measurements were carried out immediately after the preparation of the mixture and after 0.5, 1, 3, or 5 h incubation in a shaking bath.

#### Fourier transform infrared spectroscopy analysis

The samples for FTIR analysis were prepared according to the following procedure. First, the bacterial suspensions (*S. aureus* strain ATCC 6538 and *K. pneumoniae* strain ATCC 4352) were centrifuged (8000 rpm, 20 min) and washed five times with sterilized distilled water. The PS NPs stock solution was then diluted with the bacterial suspension to a concentration of 100 μg/ml and stirred for 2 h. Bacterial suspensions without the addition of PS NPs and the PS NPs stock solution diluted only with distilled water (100 μg/ml) as reference materials were also subjected to further preparation steps. All samples were frozen with liquid nitrogen and lyophilized in a freeze dryer (Christ Alpha 1–2 LD plus with the double–chamber, Osterode am Harz, Germany) for 24 h at a pressure of 0.013 mbar. The powder samples were mixed with potassium bromide (KBr), ground in an agate mortar, and pressed into KBr pellets. FTIR measurements were carried out in transmission mode using a Nicolet 6700 spectrometer (Thermo Scientific, Madison, USA). All spectra were collected at room temperature in the 4000–400 cm^−1^ with a wavenumber resolution of 4 cm^−1^.

#### Atomic force microscopy analysis

*Staphylococcus aureus* strain ATCC 6538 and *K. pneumoniae* strain ATCC 4352 grown on nutrient agar were resuspended in distilled and autoclaved water (OD_600_: ~ 0.1), followed by incubation with 2 µg/ml of PS NPs at 37 °C for 0.5, 1 and 3 h. Then, 100 µl of bacterial samples were transferred onto the mica surface previously functionalized with 0.01% Poly-l-lysine^29^. The attachment of bacterial cells to the mica surface was achieved during a 30-min incubation at 37 °C. Images of bacterial cells were collected using a NanoWizard 4 BioScience AFM (JPK/Bruker, MA, USA) operated in Quantitative Imagining (QI) mode. DNP-10 cantilevers (Bruker, MA, USA) with a nominal spring constant equal to 0.06 N/m were employed. The bacterial cells were located with an optical microscope. Then, scanning was performed to collect the topography images 3 × 3 µm (bacteria) with a resolution of 128 pixels per line. Based on the QI maps, the RMS roughness in the center of control and treated cells (from area equal 0.25 µm^2^) was determined. PS nanoparticles were also examined with NanoWizard 4 BioScience AFM microscope in QI mode (JPK/Bruker, MA, USA) in wet conditions—distilled water. Bruker SNL-10 (Bruker, MA, USA) sharp cantilevers with a nominal spring constant equal to 0.12 N/m were used for the measurements. Scanning was performed to collect the topography images 1 × 1 µm with a resolution of 128 pixels per line. Based on the topography images, PS nanoparticles size calculations were made by measuring the longest dimension of the nanoparticles.

#### Statistical analysis

Data are expressed as means ± standard deviation (SD). Differences between groups were tested for statistical significance using one-way ANOVA with Scheffe’s F test. Statistical significance was defined as p < 0.05 for all tests.

## Results and discussion

### The effect of pH and electrolyte concentration on the zeta potential of bacterial cells

The essential measurements were proceeded by an analysis of the tolerance of both bacteria used in the study to changes in the pH values of the growth environment. The tests were conducted by 24 h incubation of the bacteria on LB agar plates within a range of pH varying from pH = 3 to pH = 10. While bacteria grew well on media with pH ranged from 5 to 9, on LB agar with pH = 3 and pH = 10 their growth was significantly reduced. Therefore, in zeta potential measurements, the pH range was extended by only two units for low and high pH (from pH = 3 to pH = 1) in relation to the pH scale allowing both bacterial strains to grow.

**Figure 1:**
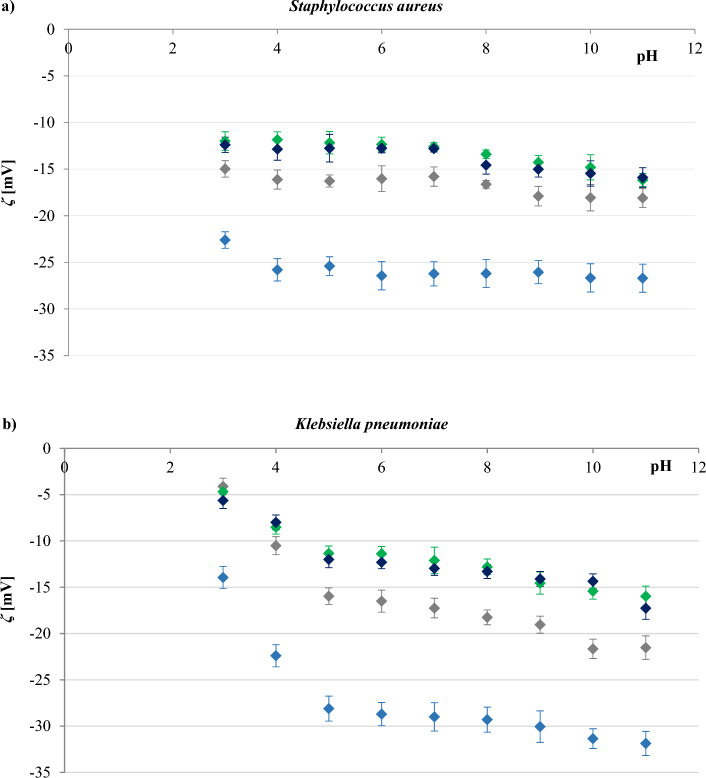
Zeta potential-pH profiles of *S. aureus* strain ATCC 6538 (**a**), and *K. pneumoniae* strain ATCC 4352 (**b**) for NaCl concentrations of (filled blue rhombus) 0.3 mM, (filled grey rhombus), 30 mM, (filled green rhombus) 100 mM, and (filled dark blue rhombus) 155 mM.

The effect of pH on the zeta potential values of *S. aureus* and *K. pneumoniae* cells in the presence of different concentrations of sodium chloride (0.3, 30, 100, and 155 mM) is shown in Fig. 1. These results clearly demonstrate, that the surfaces of the bacteria used in the study are negatively charged within the applied pH range, as in the case of most microorganisms^30^. Furthermore, the *ζ* value seems to be affected by both, pH changes as well as by sodium chloride concentration. At a constant sodium chloride concentration, the *ζ* value of *S. aureus* changed slightly with pH change, whereas for *K. pneumoniae* the value decreased significantly with increasing pH. However, as in most cells, the zeta potential of both bacterial surfaces showed a gradual increase as pH decreased due to a change in the dissociation density of functional groups present on the cell surfaces^31^. At constant pH, the *ζ* value of the cells was less negative at higher concentrations of sodium chloride. The obtained results can be explained by the compression of the double layer by counterions, such as Na^+^, the reduction of the Stern potential, and the decrease of the effective surface charge at high ionic strength^32^. It is noteworthy that all the zeta data converged into a non-zero values as the ion concentration increased (Fig. S1, Supplementary Material), indicating a spatial charge distribution on the outer surfaces of the bacteria^31^.

It could also be observed that the differences between the shapes of the four zeta potential-pH profiles obtained for each bacterium were not significant. Thus, it is evident that the concentration of the electrolyte influences only the zeta potential values, not the shape of zeta potential vs pH curves. This may indicate that an increasement in the NaCl concentration is only necessary to reduce the thickness of the double layer. The trends in the results are broadly similar to those reported by^33^ and^34^, who used a yeast suspension and different concentrations of sodium chloride during their study.

Gram-positive and Gram-negative bacteria differ significantly in their cell membrane structure and composition. The cell walls of Gram-positive bacteria are relatively simple and consist mainly of a rigid peptidoglycan backbone. On the opposite, the cell walls of Gram-negative bacteria are complex and have an outer membrane composed of lipopolysaccharides that covers a thin layer of peptidoglycan^18^. The resulting zeta potential of *K. pneumoniae* is more negative than that determined for *S. aureus* (Fig. 1), that is in line with data presented in other reports^18,35,36^. The higher negative zeta potential of their cells reflects the presence of an additional layer of negatively charged lipopolysaccharides in Gram-negative bacteria compared to Gram-positive ones.

It is widely accepted that the zeta potential is related to the stability of colloidal dispersions^37^. Colloid stability (i.e., the probability that cells will not coagulate with one another) will increase as the absolute value of the zeta potential increases. In general, a value of 25 mV (positive or negative) was taken as the arbitrary value that separates low-charged surfaces from highly-charged surfaces^38^. From the data shown in Fig. 1, we concluded that both *S. aureus* and *K. pneumonia* demonstrated good stability in 0.3 mM NaCl, so the subsequent measurements presented here were carried out at this concentration of sodium chloride.

### Nanoparticle size, polydispersity, and zeta potential

**Figure 2:**
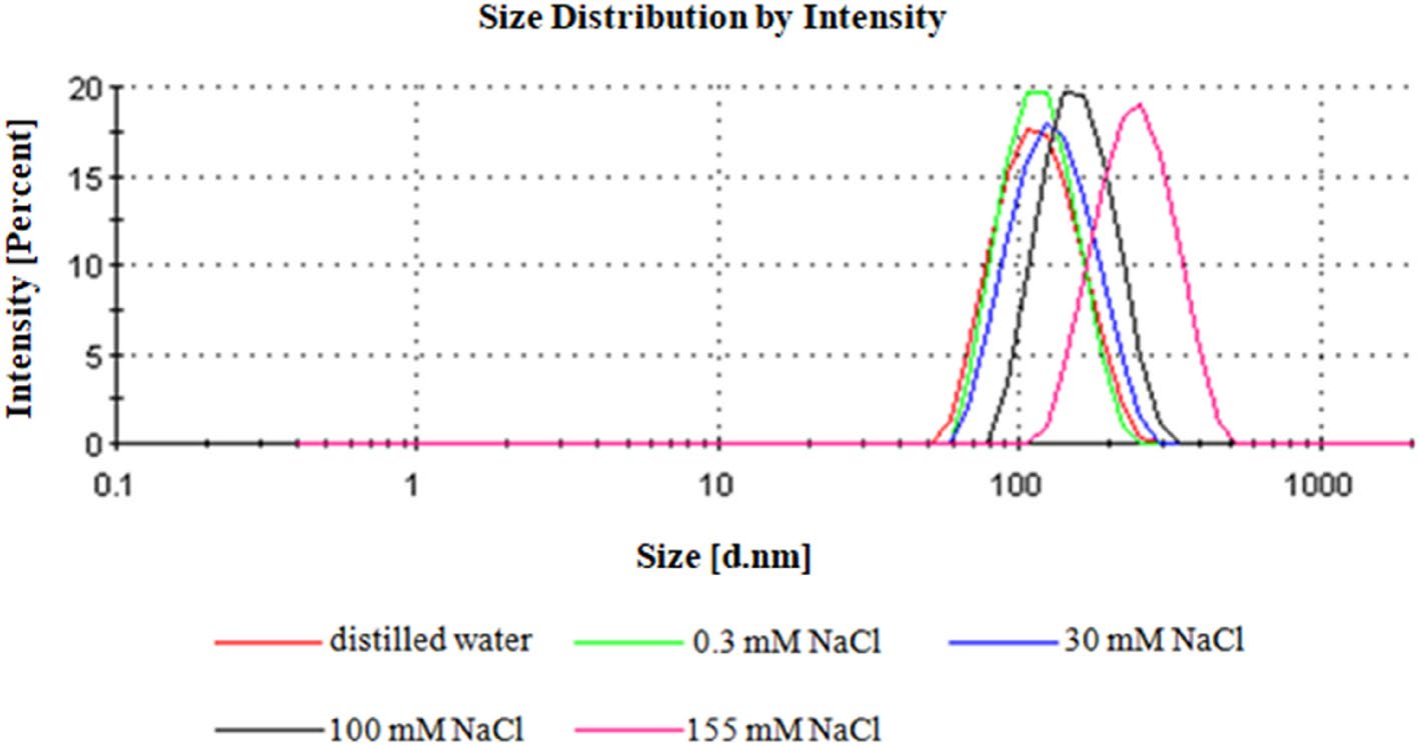
The polystyrene nanoparticles size distribution by intensity at various concentrations of NaCl.

The mean particle size, size distribution, and zeta potential are the essential parameters for establishing the quality and stability of nanoparticles. The polymer size distribution by intensity obtained by the DLS technique are summarized in Fig. 2. The hydrodynamic nanoparticle size measurements were taken in triplicate and determined values are reported along with polydispersity index (PDI) in Table 1. All samples showed mean particle diameter sizes ranging from 120.00 ± 32.61 nm to 250.50 ± 70.03 nm, with PDI ranging from 0.129 to 0.335. It was noted that on one hand the hydrodynamic particle size was affected by the sodium chloride concentration, and, on the other hand upon increasing concentration of NaCl, there was also an increase in particle size and PDI. The hydrodynamic diameter values obtained in distilled water, 0.3 and 30 mM NaCl were found to be in accordance with a nominal diameter listed by the manufacturer. Furthermore, PDI revealed monodispersed homogenous particle distribution in these three media. However, the particle size registered in 100 and 155 mM NaCl (160.80 ± 43.43 and 250.50 ± 70.03 nm, respectively) was significantly different compared to the advertised size (100 nm) and exhibited wider distribution (0.266 and 0.335, respectively). Hence, it was concluded that higher electrolyte concentrations causes interaction between the particles.

**Table 1:** Physicochemical characterization of the nanoparticles used in this study.

Medium	Hydrodynamic size [nm]	PDI	Zeta potential [mV]
0.3 mM NaCl	119.50 ± 32.02	0.050	− 48.70 ± 1.40
30 mM NaCl	133.90 ± 40.20	0.116	− 43.10 ± 4.32
100 mM NaCl	160.80 ± 43.43	0.266	− 32.40 ± 1.13
155 mM NaCl	250.50 ± 70.03	0.257	− 31.00 ± 0.99
Distilled water	120.00 ± 32.61	0.129	− 50.80 ± 2.19

**Figure 3:**
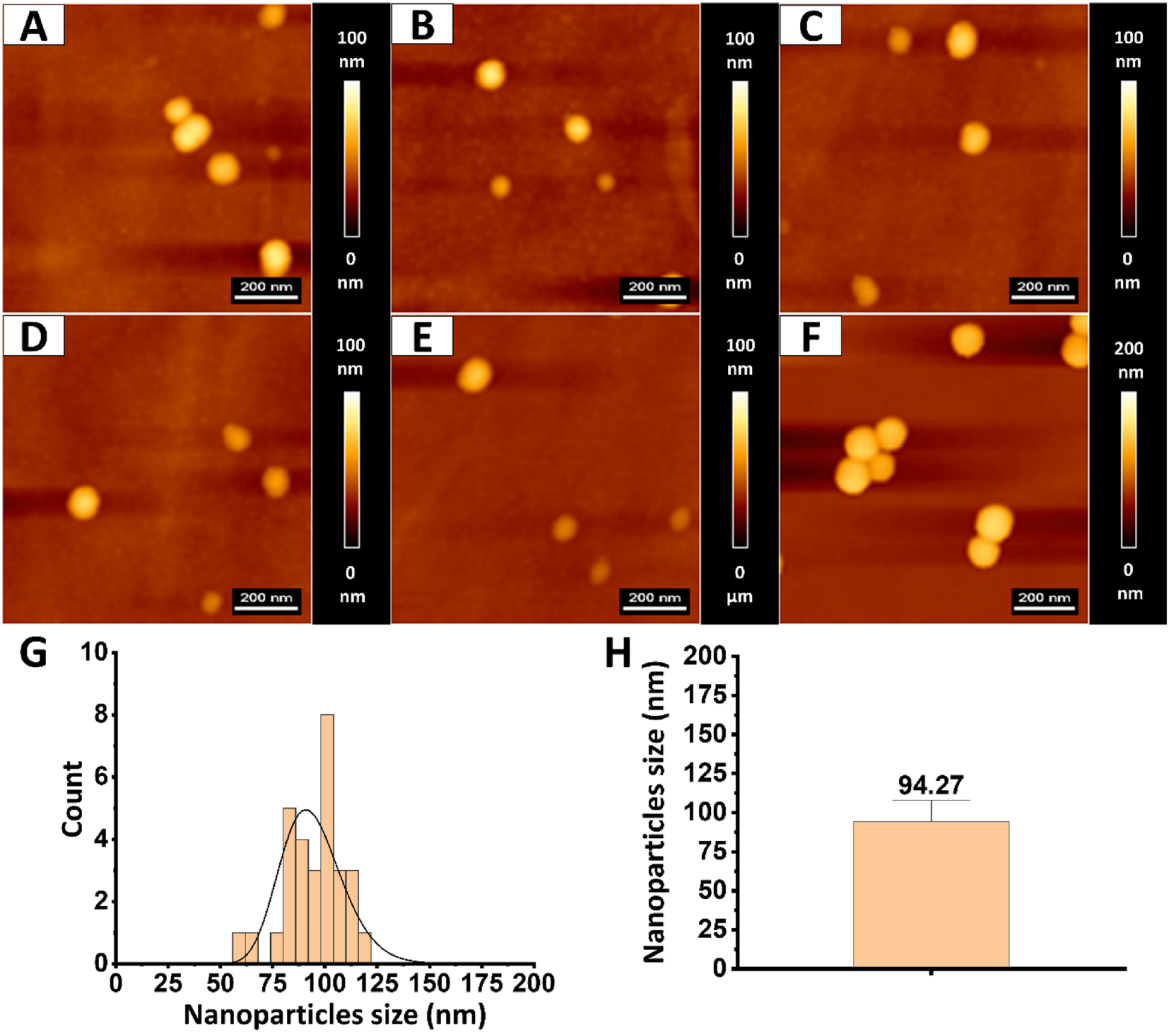
Topography and size of PS nanoparticles. (**A**–**F**) Show representative topography maps and arrangement of PS nanoparticles on the flat surface. (**G**) Shows nanoparticles size distribution measured using JPK Data Processing software. Distribution was fitted with the probability density function of the log-normal distribution. (**H**) shows particles size mean value and standard deviation.

Particle size measurements were also taken from topography maps obtained using an atomic force microscope (Fig. 3). The average size of the 30 nanoparticles converted manually was 94.27 ± 13.85 nm. The tests were conducted in distilled water. No large particle agglomerates were observed. The results confirm the data provided by the nanoparticle manufacturer about nanoparticles size and correspond with the results of the DLS method obtained in distilled water.

The stability of PS NPs was assessed by measuring the zeta potential of the particles using the ELS technique. The *ζ* values are compiled together with the hydrodynamic size and PDI in Table 1. As expected from the chemical structure of the nanoparticles, the unmodified PS NPs exhibited a negative *ζ*, indicating that these nanoparticles have a negative surface charge. However, it was also noted that particles suspended in different media possess different *ζ* values, and this may arise due to their interaction with ions present in the medium. Indeed, it was observed that when the NaCl concentration increases from 0 mM (in distilled water) to 155 mM, the *ζ* values also increase.

The subject literature shows that when the particles in suspension have a high zeta potential value (+ 30 or − 30 mV), they tend to repel each other, and there will be no tendency to form agglomerates^39–41^. Based on data collected in Table 1, it might be concluded that PS NPs are stable in distilled water, 0.3 and 30 mM NaCl, while they start to aggregate in NaCl with concentrations of 100 nM and above.

**Figure 4:**
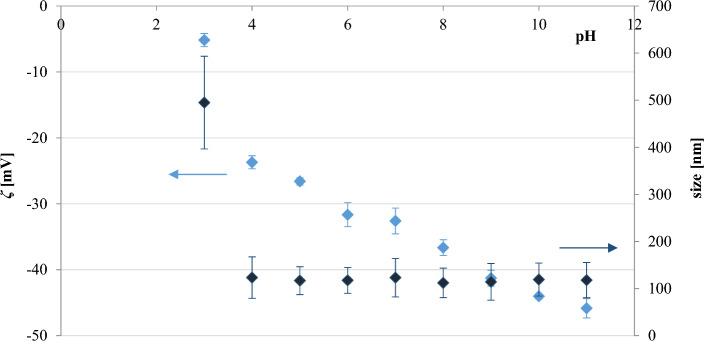
Hydrodynamic size and zeta potential values of polystyrene nanoparticles in 0.3 mM NaCl at varying pH.

The particle size and zeta potential of the polystyrene nanoparticles in 0.3 mM NaCl were then evaluated during pH titrations ranging from 3 to 11 (Fig. 4); the same range in which bacterial stability was tested (“The effect of pH and electrolyte concentration on the zeta potential of bacterial cells” section). These studies indicated that *ζ* was higher at lower pH values, reflecting the increase in the degree of protonation on the surface of the particles. However, the hydrodynamic size across the tested pH range was relatively unchanged, except for pH = 3, demonstrating the lack of particle aggregation.

All these findings reveal that an accurate combination of polymer and the medium in which this polymer is suspended, allows obtaining highly stable, narrow dispersed, and acceptable PS NPs with controlled particle size.

### Minimum inhibitory and minimum bactericidal concentrations for bacteria treated with polystyrene nanoparticles

Minimum inhibitory concentration (MIC) and minimum bactericidal concentration (MBC) assays define a test material's potency in terms of the concentration at which it inhibits the growth of (MIC) or completely kill (MBC) 99.9% microorganisms^42^.

Table 2 displays the results of testing the effect of PS NPs on MIC and MBC against *S. aureus* and *K. pneumoniae*. The MIC value of 50 mg/ml was obtained for polystyrene nanoparticles against both bacteria tested. On the contrary, MBC values against *S. aureus* and *K. pneumoniae* could not be observed, which was not unexpected since a positive charge of the particles is required for toxicity against bacterial cells^43^. Thus, it can be concluded that 100 nm PS NPs can slow down the growth of the tested bacteria but cannot kill them within the evaluated concentrations.

**Table 2:** Determination of minimal inhibitory concentration (MIC) and minimal bactericidal concentration (MBC) for bacteria treated with PS nanoparticles.

Bacteria strain	PS NPs concentration [mg/ml]	
	MIC	MBC^a^
*Staphylococcus aureus* strain ATCC 6538	50	N.O
*Klebsiella pneumoniae* strain ATCC 4352	50	N.O

^a^N.O., not observed.

**Figure 5:**
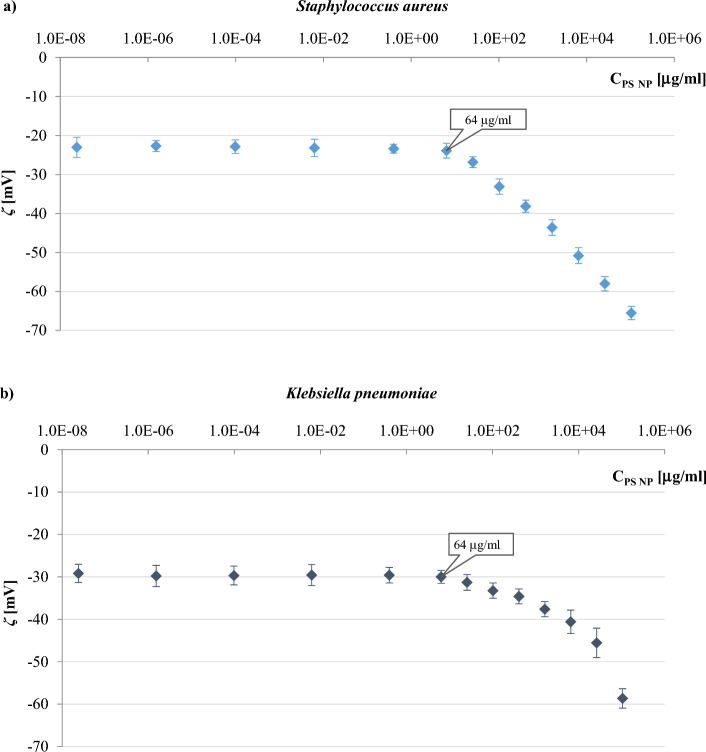
The zeta potential-pH profiles in 0.3 mM NaCl obtained for *S. aureus* strain ATCC 6538 (**a**), and *K. pneumoniae* strain ATCC 4352 (**b**) incubated overnight with different concentrations of polystyrene nanoparticles.

Further, suspensions of both bacteria incubated with PS NPS for 24 h were used to determine the zeta potential of the samples in 0.3 mM NaCl (Fig. 5). Interestingly, lower concentrations of nanoparticles (i.e., up to 64 µg/ml) did not show a meaningful effect on the *ζ* value of both bacterial cells used in the study. The presence of negative potentials on both PS NP_S_ and bacterial surfaces prevented the direct interaction between the nanoparticles and the bacteria due to the prevailing electrostatic repulsion at the interface. In contrast, PS concentrations higher than 64 µg/ml significantly affected the zeta potential of bacterial cells. The molecular crowding upon increasing the nanoparticles concentration supports this outcome, resulting in a net interactive interaction at the nanoplastic—bacteria interface.

PS likely attaches to the cell surface, blocks positively charged sites on the bacteria surface, and then increases the available negatively charged groups^25^, which in turn leads to a reduction in the rate of bacterial growth, but without killing bacteria as determined in MIC and MBC tests. It may also be stated that a significant increase in negative *ζ* values with increasing PS concentration is not related to bacterial death as the zeta potential of dead cells is less negative than those values determined for living cells^22,25^.

The nanoparticles, depending on their surface chemistry, charge and hydrophobicity, interact with lipids, polysaccharides or proteins of the bacterial cell membrane^44^. Nanoparticles can interact with structural proteins, membrane proteins and enzymes through electrostatic, hydrophobic, hydrogen-bonding or Van der Waals interactions. Electrostatic, hydrophobic, hydrogen bonding and electrosteric repulsion interactions can occur between polysaccharides and nanoparticles, whereas hydrophobic and electrostatic interactions are indicated between lipids and nanoparticles^45^. Perini et al.^46^ reported that 60 nm nonfunctionalized polystyrene, aminated polystyrene, and carboxylated polystyrene are able to induce membrane permeability without leading to membrane disintegration. The conservation of membrane integrity, a crucial issue to distinguish between particles binding and internalization in cells, is consistent with the previous findings for the interaction between PS particles of different sizes (from 20 nm to 2 μm) and giant unilamellar vesicles. Ning et al.^47^ investigated the interaction between bacteria and PS NPs. The results demonstrated that 30 nm, 200 nm and amino-modified PS particles adsorbed onto the bacterial surface. The positively charged amino-modified PS particles showed more interaction with the bacterial cells. These particles exerted their antibacterial effect mainly by increasing intracellular ROS levels in bacteria, whereas 30 nm PS acted on bacteria through a combination of ROS generation and mechanical damage. It was suggested that the 30 nm PS particles may penetrate bacterial membranes and disrupt membrane protein functions, possibly entering bacterial cells and disrupting their function.

### The effect of polystyrene nanoparticles concentration on the zeta potential of bacteria cells

Based on Fig. 5, the nanoparticle concentrations of 0.4, 2, 20, and 100 μg/ml, were selected for further microelectrophoretic studies. The first three concentrations were chosen from a range of concentrations in which both bacteria incubated overnight with PS NPs possess a constant value. The final concentration was in the range of concentrations that changed the zeta potential value after long-term treatment of the bacteria with nanoparticles, but this concentration was still far from the upper limit of environmentally relevant concentration (250 µg/ml)^48–50^.

**Figure 6:**
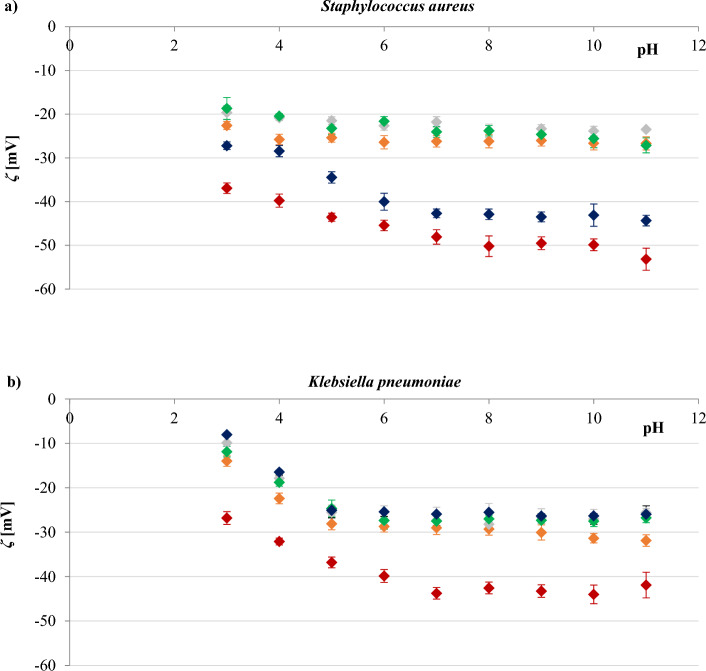
The zeta potential-pH profiles in 0.3 mM NaCl obtained for *S. aureus* strain ATCC 6538 (**a**), and *K. pneumoniae* strain ATCC 4352 (**b**). Bacterial cells were untreated (filled orange rhombus) or treated with 0.4 (filled grey rhombus), 2 (filled green rhombus), 20 (filled dark blue rhombus), and 100 (filled brown rhombus) µg/ml of polystyrene nanoparticles. Statistical analysis is presented in Tables S1 and S2 of the Supplementary Material.

The effect of PS NPs concentration on the zeta potential of *S. aureus* and *K. pneumoniae* strains was studied using samples prepared by thoroughly mixing the nanoparticles and bacterial suspension 20 min before measurements. The resulting *ζ* values determined at varying pH plotted against particle concentration are depicted in Fig. 6.

Data presented in Fig. 6 pointed out that mixing *S. aureus* cells with polystyrene particles at final concentrations of 0.4 and 2 µg/ml resulted in slightly less negative *ζ* values than the control sample (bacteria without PS NPs). An analogous response in the alteration of the zeta potential value was observed for samples with *K. pneumoniae* and polystyrene particles at a concentration of 0.4, 2, or 20 µg/ml. The observed changes can be explained by assuming the probable attachment of PS to the cell surface and the resulting shielding of the surface charge. A similar hypothesis has been proposed to explain the interactions between negatively charged titanium dioxide nanoparticles and rhizosphere bacteria^24^ as well as neutral polyethylene oxide particles and *E. coli* and *S. aureus* bacteria^25^. It is also likely that the attached nanoparticles may attract ions from the surrounding buffer. The zeta potential of negatively charged PS NPs is influenced by three competing processes occurring due to the combined actions of ions of the same and opposite charge surrounding the particles: (1) neutralisation of the negative charge on the surface by adsorption of the opposite charge ions leading to a reduction of the *ζ*; (2) the same charge ions drawing near to the hydrophobic surface of the molecules increasing the *ζ*; (3) the diffusive double layer’s compression caused by the high mass concentration of the electrolyte, resulting in drops of the *ζ*^51^. However, the attachment of PS to the cell surface is relatively weak and tends to be reversible since it is mainly determined by Van der Waals attraction and double-layer repulsion^52^. The *K. pneumoniae* cells, having a relatively thin cell wall additionally surrounded by a membrane containing fatty substances and polysaccharides, enable the attachment of more PS molecules so that the attachment was also observed when nanoparticles of 20 µg/ml were used.

In contrast, a significant increase in negative *ζ* values over the tested pH range was observed after the treatment of *S. aureus* with nanoparticles at concentrations of 20 or 100 µg/ml and when *K. pneumoniae* were treated with nanoparticles at a concentration of 100 µg/ml. Therefore, it can be concluded that PS NP_S_ block only positively charged sites on the bacterial surface, thus increasing the available negatively charged groups.

### The effect of exposure time to polystyrene nanoparticles on the zeta potential of bacterial cells

Subsequent studies focused on measuring the zeta potential of *S. aureus* and *K. pneumoniae* as a function of pH depending on the exposure time of the bacteria to PS NPs. Experimental research was performed immediately after suspending the bacterial cells in a 0.3 mM NaCl solution containing the polystyrene at a concentration of 20 μg/ml, and after the 1 h and 3 h exposure of the bacterial cells to the nanoparticles. This concentration was chosen based on the data presented in Fig. 6, considering the completely contrary effect on *ζ* values of the cells.

**Figure 7:**
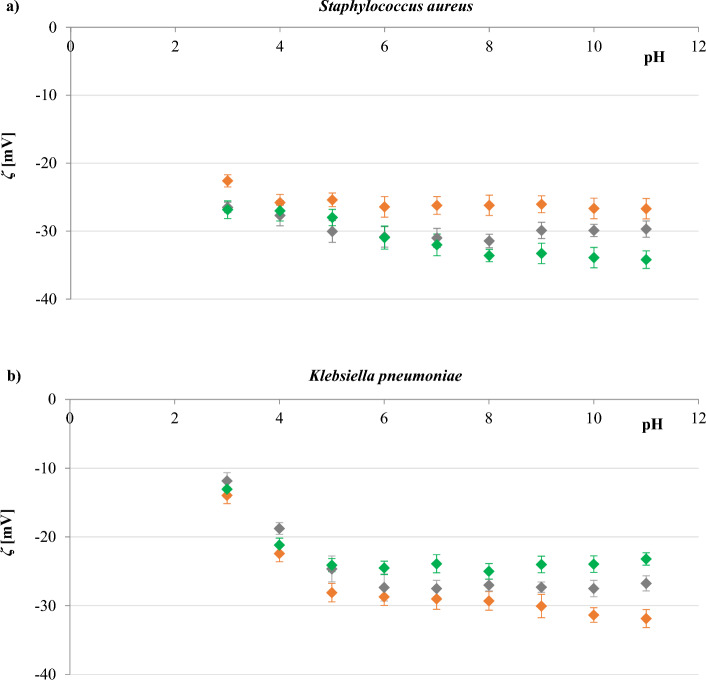
The zeta potential-pH profiles in 0.3 mM NaCl obtained for *S. aureus* strain ATCC 6538 (**a**), and *K. pneumoniae* strain ATCC 4352 (**b**). Zeta potential values were measured directly (filled orange rhombus), after 1 h (filled grey rhombus), and 3 h (filled green rhombus) of exposure of bacteria to the polystyrene nanoparticles with a concentration of 20 μg/ml. Statistical analysis is presented in Tables S3 and S4 of the Supplementary Material.

The dependence of the zeta potential as a function of pH for analysed bacteria is presented in Fig. 7. As observed, in the case of *S. aureus*, more negative zeta potential values occurred alongside increased the exposure time. Conversely, in the case of *K. pneumoniae*, less negative potential values were obtained as exposure time increased.

The zeta potential values obtained for all tested PS NP concentrations (0.4, 2, 20, and 100 μg/ml) and all periods (0, 0.5, 1, 3, and 5 h) at the three selected pHs are summarized in Table S1 (Supplementary Material). The observed variation in *ζ* measured after different exposure times of bacteria to the nanoparticles can be explained by the possibility of the fractional settlement of the particles in dispersion (either bacteria, NPs or aggregates) during performed experiment. Zeta potential values obtained after 3 and 5 h of treatment of the bacteria with polystyrene particles are very close to each other, indicating the saturation of cell surfaces. However, constant zeta values were not obtained with increasing particle concentration, as demonstrated by Lyden et al.^53^ during the characterization of carboxylate nanoparticle adhesion with fungi *Candida albicans*. These authors achieved the nanoparticle isotherms illustrating Langmuir-like behavior, reflecting an increase in nanoparticle binding with their increasing concentrations, up to the complete cell wall saturation. These isotherms enabled an understanding of the adsorption behavior using a simple model and an estimate of the adsorption binding energy.

The cell surfaces of *S. aureus* and *K. pneumoniae* are negatively charged, and the electrostatic interaction acts as a repulsive force in the adhesion of negatively charged polystyrene particles to these surfaces. However, bacterial surfaces possess acidic and basic functional groups, which influence the electrostatic behavior of the cells, thus regulating the probability of polystyrene nanoparticles adhesion on bacteria. In this respect, the PS surface containing styrene groups can adhere to bacteria through electrostatic and van der Waals interactions^54^.

Since the zeta potential is determined by the electrophoresis of a charged particle in a liquid medium, it represents the net charge of the electric double layer around the particle. The zeta potential is therefore not directly indicative of the surface characteristics of the particle, which means that it is unlikely to be involved in direct adhesion interactions between cells and nanoparticles. However, the zeta potential of a molecule probably plays a role in the adhesion of a charged surface to bacteria, primarily through a long-range effect^55^.

### Fourier transform infrared spectroscopy (FTIR) evaluation of the interaction between bacteria cells and PS NPs

**Figure 8:**
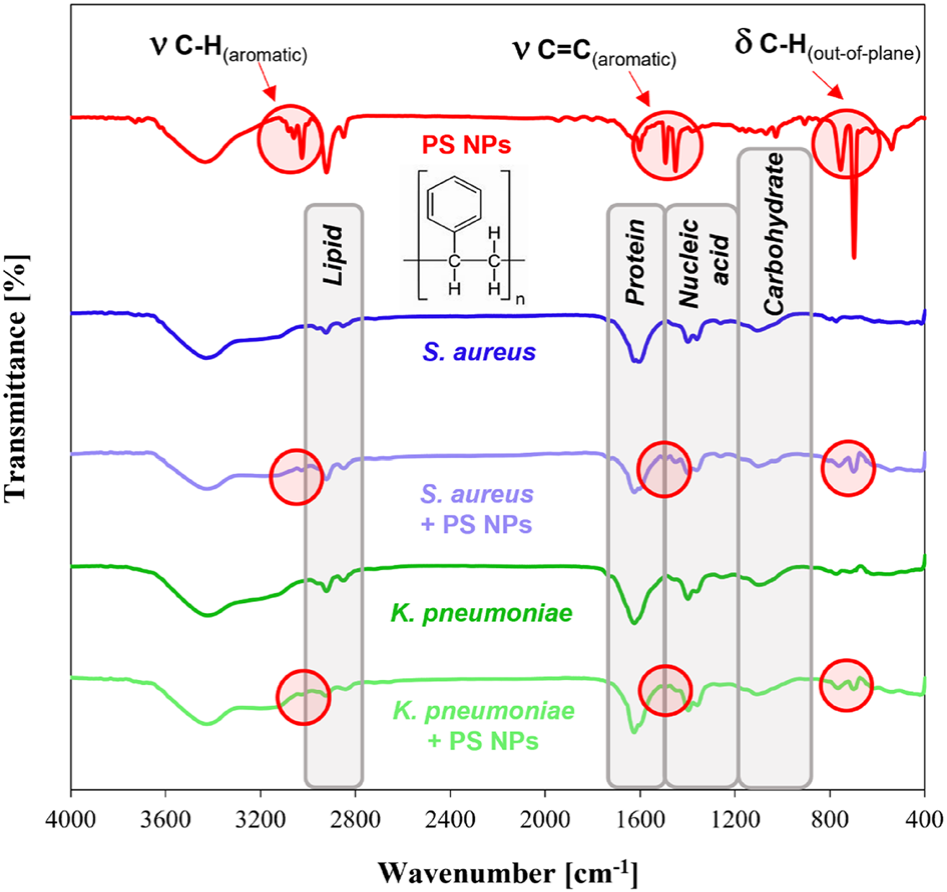
FTIR spectra collected for polystyrene nanoparticles and bacteria (*S. aureus* strain ATCC 6538, and *K. pneumoniae* strain ATCC 4352) before treatment (dark blue and dark green lines, respectively) and after treatment with polystyrene nanoparticles (100 µg/ml) (light blue and light green lines, respectively).

Identification or discrimination of bacteria is more and more often investigated using FTIR spectroscopy due to the strong correlation between the position of absorption bands in the recorded spectra and the biological information about the analyzed microorganisms^56,57^. Specific signals observed in the FTIR spectra collected for bacteria indicate the presence of organic compounds that build their cells^56^. The classification proposed by Naumann et al.^56,58^ distinguishes four spectral subranges corresponding to particular groups of compounds, namely lipids (vibrations of functional groups typical for fatty acids in the range of 3000–2800 cm^−1^), proteins (vibrations of amide bands in the range of 1800–1500 cm^−1^), nucleic acids (1500–1200 cm^−1^), and carbohydrates (1500–1200 cm^−1^), as well as the fingerprint region with a unique spectral pattern not assigned to the essential cellular components (900–700 cm^−1^). All these regions are observed in the spectra collected for *S. aureus* and *K. pneumoniae*, shown in Fig. 8 (dark blue and dark green lines, respectively).

The FTIR spectra of selected bacteria recorded after their treatment with 100 nm polystyrene particles (Fig. 8, light blue and light green lines) showed the same bands with similar intensity as the spectra of reference materials (Fig. 8, dark blue and dark green lines). This proves that the interaction of the studied nanoparticles with the surface of *S. aureus* and *K. pneumoniae* is not destructive and does not cause significant changes in the structure of the bacteria. However, it should be emphasized that the presence of PS NPs on the surface of the tested bacteria was confirmed by the appearance of bands characteristic for PS in their spectra (Fig. 8, light blue and light green lines vs. the red line). The low intensity signals at 3025–3035 cm^−1^ were assigned to the C–H stretching vibration in the benzene rings, while the weak bands at 1608, 1499, and 1457 cm^−1^ corresponded to the aromatic C=C stretching vibration in the polystyrene structure. The bands in the fingerprint region at around 754 and 684 cm^−1^ were, in turn, related to C–H out-of-plane bending vibrations^59^. The position of these bands in recorded spectra (Fig. 7, light blue and light green lines) is very close to the position of corresponding signals in the reference spectrum of the polystyrene (Fig. 8, red line). Therefore, treating *S. aureus* and *K. pneumoniae* with 100 nm PS does not affect the band shift in their spectra, indicating functional groups' stability in the bacterial structure under given conditions.

### Atomic force microscopy (AFM) evaluation of the interaction between bacteria cells and PS NPs

As a scanning probe technique, AFM is ideally suited for investigating the surface properties of bacteria, including topography, composition, and adhesion^60^. In addition, the capacity of AFM to capture high-resolution, a capability once exclusive to electron microscopy, facilitates quality imaging of nanoscale structures. In contrast to electron microscopy, AFM sample preparation does not require fixation and dehydration, which allows for the imaging of live cells in a native state. Acknowledging these advantages, we used AFM to investigate the potential interaction of PS NPs with bacterial structures immobilized on surfaces in an aqueous environment to understand polystyrene nanoparticles' environmental behavior better.

**Figure 9:**
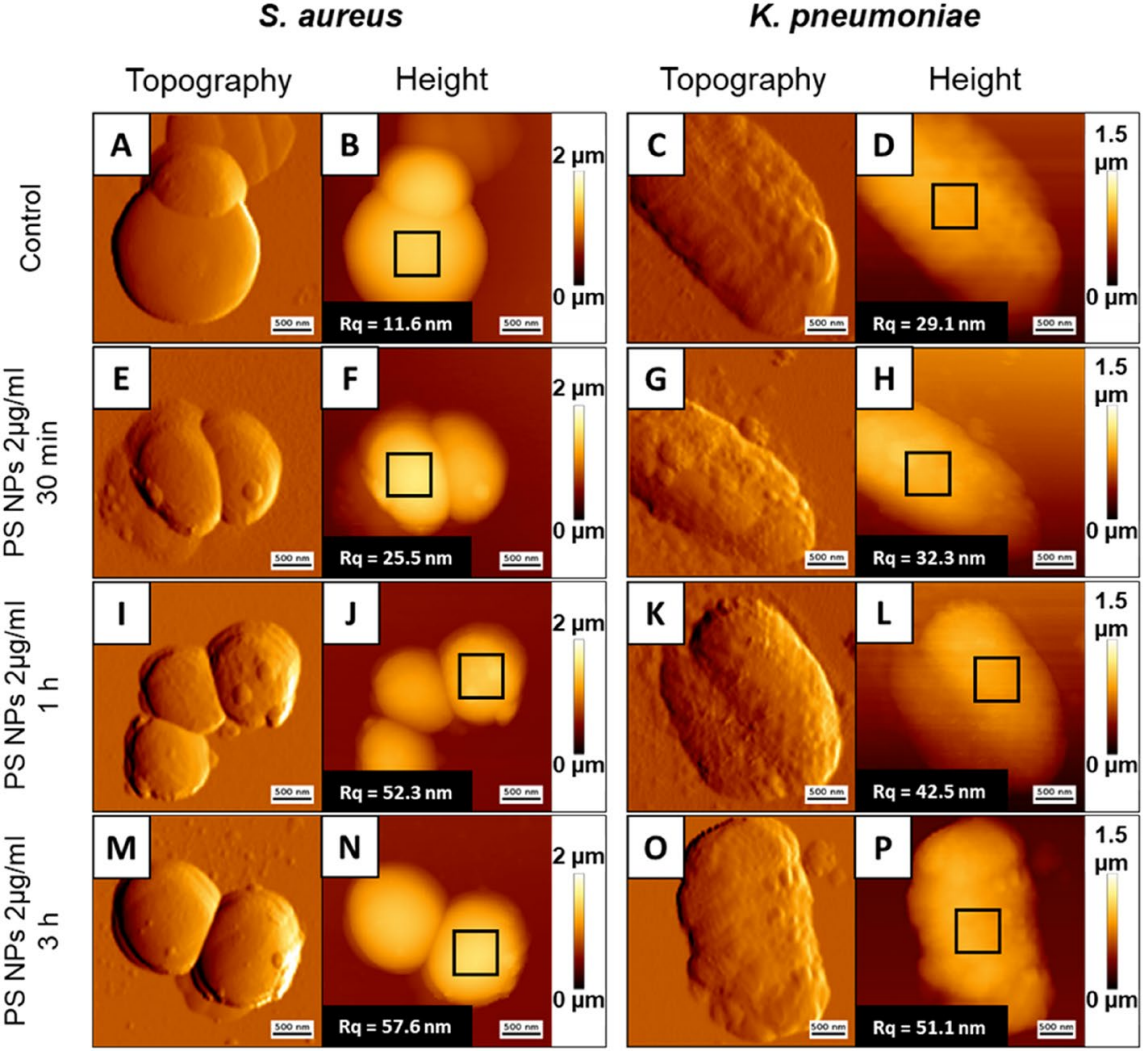
Surface topography (**A**,**C**,**E**,**G**,**I**,**K**,**M**,**O**) and height images (**B**,**D**,**F**,**H**,**J**,**L**,**N**,**P**) of *S. aureus* strain ATCC 6538, and *K. pneumoniae* strain ATCC 4352 exposed to 100 nm polystyrene nanoparticles at doses of 2 µg/ml for 0.5 h (**E**–**H**), 1 h (**I**–**L**), and 3 h (**M**–**P**) compared to untreated control (**A**–**D**). Scale bar—500 nm. Inside the panels the marked areas (black squares) from which the surface RMS roughness were calculated and roughness values (Rq parameter) of measured bacteria surfaces (root mean square average of the profile heights over the evaluation length).

The *S. aureus* and *K. pneumoniae* cells were treated with 2 µg/ml of PS NPs three times (0.5, 1, and 3 h) before being immobilized on the mica surface and visualized using AFM. As evidenced, both tested bacteria were stably imaged for at least 3 h (Fig. 9). The presence of PS NPs on bacterial surfaces was seen in both samples, suggesting the particles' affinity for bacterial structures. These PS NPs did not form aggregates, peel off, or dissolve in water during or after measurements. Furthermore, AFM analysis showed no significant change in the morphology of the treated bacteria. After exposure to the nanoparticles, there was no decrease in the height of the samples tested, which would indicate a breakdown of the bacterial cell structure. Surface roughness measurements also showed no significant changes in cell surface quality after treatment. The increase in surface roughness during incubation may be due to the presence of nanoparticles on the cell surface. Their number may have increased with increasing incubation time, which resulted in increased roughness. These results confirm that 100 nm PS NPs, at least at low concentrations, cannot kill *S. aureus* and *K. pneumonia*.

### Bacterial cell size measurements

The determination of cell size is a fundamental challenge for all living organisms. Under given growth conditions, the cell size of bacterial species usually falls within a narrow range. However, cell size can vary widely between species, shaped by nutritional, mechanical, and genetic factors^61^. In addition, heterogeneities in cell size within a given bacterial cell population, and differences in the size of a single bacterium depending on growth conditions, have also been found^22^.

According to the subject literature, *S. aureus* bacteria are approximately 0.5–1.5 μm in diameter and divide in more than one plane to form irregular, three-dimensional clusters of cells described as “grape-like”. Individual colonies on nonselective media are smooth, round, convex, and glistening and may reach a size of 4–6 mm in diameter with a sharp border^62,63^. However, *K. pneumoniae* is a straight rod-shaped bacterium, 0.3–1.0 μm in diameter and 0.6–6.0 μm in length. The rods are arranged singly, in pairs, or short chains^64^.

Images obtained by AFM (Fig. 9) revealed that *S. aureus* is a spherical bacterium, while *K. pneumoniae* cell exhibits a rod shape. The images also make it possible to determine the size of the two bacteria studied, which are gathered in Table S6. The DLS technique also assessed the size of *S. aureus* and *K. pneumoniae* cells. The plots of the bacterial size distribution by intensity registered at 0.3 mM NaCl are shown in Fig. S2, together with the plot obtained for PS NPs. In addition, the average hydrodynamic diameters are collected in Table S6.

Electron microscopy measurements of the structural cell surface features aim to determine the length of these structures^65^, but these studies are often very tedious. Dynamic light scattering is one such technique that helps overcome these obstacles. Assuming that the maximum variation in hydrodynamic radius observed as a function of pH represents the maximum length of surface structures, DLS can be an alternative method for determining the length of cells. The values summarized in Table S6 confirm that AFM and DLS can successfully determine the size of *S. aureus* and *K. pneumoniae* and it is possible to compare particle sizes obtained by both of the measuring techniques. However, it should be borne in mind that since DLS measures the hydrodynamic radius of particles, the size obtained is generally larger than that obtained by AFM.

## Conclusions

Nowadays, a rapid increase in plastic production (including PS) has been noted worldwide. This plastic, being released into the environment in the form of micro- and nanoparticles, is the observed in both industrialized and natural areas. Despite the lack of literature resources concerning environmental occurrence of nanoplastics, one might rightfully expect their presence in the environment, with greater quantity than bigger in size microplastics. Thus, nanoplastics are considered more toxic than microplastics due to their ability to permeate cell membranes and disrupt cell functions.

In this study, the effect of interaction between 100 nm polystyrene nanoparticles and *Staphylococcus aureus* and *Klebsiella pneumoniae*, on the surface potential (represented as zeta potential) of the bacteria was examined. It was shown that PS NPs caused a change in the zeta potential value of both strains, depending on particle concentration, pH, and also on the time of the bacteria’ exposure to them. The nanoparticles were claimed to attach to the cell membranes of bacteria, shifting their electrical charge, which in turn leads to a reduction in the rate of bacterial growth, but without killing bacteria as determined in MIC and MBC tests. Through the application of atomic force microscopy and Fourier transform infrared spectroscopy, the presence of PS NPs on bacterial surfaces was detected on the bacterial cells.

From the experimental results of the present paper, it might be concluded that measuring the zeta potential is a valuable approach for a deeper understanding of the critical relationship between nanoparticles and cells. Being rapid, straightforward, and inexpensive, the DLS technique can be adopted to screen particles with properties that alter the surface charge of biological membranes.

It is crucial to emphasize that the interactions between all plastic particles and the environment are not explainable through a single nanoplastic model. Many variables exist that influence the PS NPs interaction with cells and their way of entering the cells. With the diversity of nanoparticle applications and even broader potential in the future, understanding and predicting their behaviour and effects on biological systems is becoming extremely important.

## Supplementary Information

Supplementary Information.

## Data Availability

The data sets supporting our conclusions are presented in the article.

## Abbreviations

AFM: Atomic force microscopy
ATCC: American Type Culture Collection
DLS: Dynamic light scattering
ELS: Electrophoretic light scattering
FTIR: Fourier transform infrared spectroscopy
MIC: Minimum inhibitory concentration
MBC: Minimum bactericidal concentration
PDI: Polydispersity index
PS NPs: Polystyrene nanoparticles
*ζ*: Zeta potential (electrokinetic potential)
