# Supplementary material for: Exposure to polystyrene nanoparticles leads to changes in the zeta potential of bacterial cells
Source: Sci Rep. 2023 Jun 12;13:9552. doi: 10.1038/s41598-023-36603-5 (PMC10260929; doi:10.1038/s41598-023-36603-5)
Supplement: Supplementary file 1 — Supplementary Information. [file 41598_2023_36603_MOESM1_ESM.docx]

**Exposure to polystyrene nanoparticles leads to changes in the zeta potential of bacterial cells**

Marcin Zając^a^, Joanna Kotyńska^b^, Grzegorz Zambrowski^c,d^, Joanna Breczko^e^, Piotr Deptuła^f^, Mateusz Cieśluk^f^, Monika Zambrzycka^c^, Izabela Święcicka^c,d^, Robert Bucki^f^, Monika Naumowicz^b,*^

*^a^ Doctoral School of Exact and Natural Sciences, University of Bialystok, 1K K. Ciolkowski Str., 15-245 Bialystok, Poland; m.zajac@uwb.edu.pl*

*^b^ Laboratory of Bioelectrochemistry, Department of Physical Chemistry, Faculty of Chemistry, University of Bialystok, 1K K. Ciolkowski Str., 15-245 Bialystok, Poland; joannak@uwb.edu.pl, monikan@uwb.edu.pl*

*^c^ Laboratory of Molecular Biophysics, Department of Microbiology and Biotechnology, Faculty of Biology, University of Bialystok, 1J K. Ciolkowski Str., 15-245 Bialystok, Poland; g.zambrowski@uwb.edu.pl, m.zambrzycka@uwb.edu.pl, izabelas@uwb.edu.pl*

*^d^ Laboratory of Applied Microbiology, Department of Microbiology and Biotechnology, Faculty of Biology, University of Bialystok, 1J K. Ciolkowski Str., 15-245 Bialystok, Poland.*

*^e^ Laboratory of Materials Chemistry, Department of Physical Chemistry, Faculty of Chemistry, University of Bialystok, 1K K. Ciolkowski Str., 15-245 Bialystok, 15-245 Bialystok, Poland;* [*j.luszczyn@uwb.edu.pl*](mailto:j.luszczyn@uwb.edu.pl)

*^f^ Department of Medical Microbiology and Nanobiomedical Engineering, Medical University of Bialystok, 2C A. Mickiewicz Str., 15-222 Bialystok, Poland;* *piotr.deptula@umb.edu.pl, mticv1@gmail.com, buckirobert@gmail.com*

**

Fig. S1. Potential zeta measurements of bacterial cells as a function of ionic concentration at pH = 7.4. Statistical significance was defined as * p < 0.05; ** p < 0.01.

Tab. S1. The zeta potential of *S. aureus* bacterial cells after exposure to polystyrene nanoparticles PS NPs (C = 0.4, 2, 20, and 100 µg/ml).

| pH | ***S. aureus*** | | | | |
| --- | --- | --- | --- | --- | --- |
|  | control | 0.4 µg/ml | 2 µg/ml | 20 µg/ml | 100 µg/ml |
| 3.0 | -22.60 ± 0.89 | -19.65 ± 1.62^a^ | -18.70 ± 2.51 | -27.23 ± 0.86^b,c^ | -36.93 ± 1.20^a,b,c,d^ |
| 4.0 | -25.80 ± 1.20 | -20.83 ± 0.64^a^ | -20.45 ± 0.57^a^ | -28.45 ± 1.29^a,b,c^ | -39.78 ± 1.54^a,b,c,d^ |
| 5.0 | -25.40 ± 1.00 | -21.50 ± 0.88^a^ | -23.25 ± 1.45 | -34.48 ± 1.31^a,b,c^ | -43.58 ± 0.93^a,b,c,d^ |
| 6.0 | -26.43 ± 1.51 | -22.65 ±1.02^a^ | -21.65 ± 1.07 | -40.03 ± 1.94^a,b,c^ | -45.45 ± 1.20^a,b,c,d^ |
| 7.0 | -26.23 ±1.30 | -21.80 ± 1.20^a^ | -24.05 ± 1.23 | -42.70 ± 1.05^a,b,c^ | -48.08 ± 1.65^a,b,c,d^ |
| 8.0 | -26.20 ± 1.51 | -23.83 ± 1.53^a^ | -23.80 ± 1.21 | -42.90 ± 1.20^a,b,c^ | -50.20 ± 2.34^a,b,c,d^ |
| 9.0 | -26.05 ± 1.24 | -23.35 ± 0.95^a^ | -24.65 ± 1.33 | -43.50 ± 1.13^a,b,c^ | -49.53 ± 1.46^a,b,c,d^ |
| 10.0 | -26.67 ± 1.54 | -23.85 ± 1.06^a^ | -26.60 ± 2.07 | -43.10 ± 2.55^a,b,c^ | -49.88 ± 1.34^a,b,c,d^ |
| 11.0 | -26.70 ± 1.59 | -23.50 ± 0.93^a^ | -27.15 ± 1.71 | -44.35 ± 1.15^a,b,c^ | -53.18 ± 2.52^a,b,c,d^ |

^a^ Statistically significant differences vs. control group, p < 0.05. ^b^ Statistically significant differences vs. modified membranes with PS NPs (C = 0.4 µg/ml ), p < 0.05. ^c^ Statistically significant differences vs. modified membranes with PS NPs (C = 2 µg/ml ), p < 0.05. ^d^ Statistically significant differences vs. modified membranes with PS NPs (C = 20 µg/ml ), p < 0.05.

Tab. S2. The zeta potential of *K. pneumoniae* bacterial cells after exposure to polystyrene nanoparticles PS NPs (C = 0.4, 2, 20, and 100 µg/ml).

| pH | ***K. pneumoniae*** | | | | |
| --- | --- | --- | --- | --- | --- |
|  | control | 0.4 µg/ml | 2 µg/ml | 20 µg/ml | 100 µg/ml |
| 3.0 | -13.95 ± 1.20 | -9.86 ± 0.28^a^ | -8.02 ± 0.44^a^ | -11.85 ± 1.20^c^ | -26.80 ± 1.43^a,b,c,d^ |
| 4.0 | -22.40 ± 1.20 | -17.85 ± 0.71^a^ | -16.45 ± 0.51^a^ | -18.78 ± 0.85^a^ | -32.10 ± 0.78^a,b,c,d^ |
| 5.0 | -28.10 ± 1.35 | -25.50 ± 1.27^a^ | -25.05 ± 1.16 | -24.65 ± 1.89 | -36.80 ± 0.76^a,b,c,d^ |
| 6.0 | -28.70 ± 1.25 | -27.40 ± 0.97^a^ | -25.40 ± 1.33 | -27.35 ± 1.97 | -39.85 ± 1.44^a,b,c,d^ |
| 7.0 | -29.00 ± 1.53 | -27.35 ± 1.12^a^ | -25.90 ± 1.55 | -27.50 ± 1.20 | -43.75 ± 1.31^a,b,c,d^ |
| 8.0 | -29.30 ± 1.35 | -28.20 ± 1.34^a^ | -25.50 ± 1.97 | -27.00 ± 0.85 | -42.55 ± 1.32^a,b,c,d^ |
| 9.0 | -30.05 ± 1.70 | -26.55 ± 0.71^a^ | -26.35 ± 1.64 | -27.30 ± 0.75 | -43.25 ± 1.41^a,b,c,d^ |
| 10.0 | -31.35 ± 1.06 | -27.00 ± 1.23^a^ | -26.30 ± 1.45 | -27.50 ± 1.20 | -44.00 ± 2.10^a,b,c,d^ |
| 11.0 | -31.87 ± 1.31 | -25.60 ± 1.55^a^ | -25.95 ± 1.28 | -26.75 ± 1.10 | -41.90 ± 2.89^a,b,c,d^ |

^a^ Statistically significant differences vs. control group, p < 0.05. ^b^ Statistically significant differences vs. modified membranes with PS NPs (C = 0.4 µg/ml ), p < 0.05. ^c^ Statistically significant differences vs. modified membranes with PS NPs (C = 2 µg/ml ), p < 0.05. ^d^ Statistically significant differences vs. modified membranes with PS NPs (C = 20 µg/ml ), p < 0.05.

Tab. S3. The zeta potential of *S. aureus* bacterial cells after exposure to polystyrene nanoparticles PS NPs (20 µg/ml). Tests were carried out immediately after sample preparation (marked as control), and after 1 and 3 hours.

| pH | ***S. aureus*** | | |
| --- | --- | --- | --- |
|  | control | 1h | 3h |
| 3.0 | -22.60 ± 0.89 | -26.85 ± 1.30^a^ | -26.50 ± 0.76^a^ |
| 4.0 | -25.80 ± 1.20 | -27.00 ± 1.50^a^ | -27.70 ± 1.53^a,b^ |
| 5.0 | -25.40 ± 1.00 | -28.00 ± 1.20^a^ | -30.05 ± 1.60^a,b^ |
| 6.0 | -26.43 ± 1.51 | -30.95 ± 1.70^a^ | -30.85 ± 1.50^a^ |
| 7.0 | -26.23 ±1.30 | -32.03 ± 1.60^a^ | -31.00 ± 1.40^a^ |
| 8.0 | -26.20 ± 1.51 | -33.60 ± 0.90^a^ | -31.45 ± 1.00^a^ |
| 9.0 | -26.05 ± 1.24 | -33.90 ± 1.50^a^ | -29.90 ± 1.20^a^ |
| 10.0 | -26.67 ± 1.54 | -33.29 ± 0.90^a^ | -29.90 ± 0.90^a^ |
| 11.0 | -26.70 ± 1.59 | -34.20 ± 1.30^a^ | -29.70 ± 1.20^a^ |

^a^ Statistically significant differences vs. control group, p < 0.05. ^b^ Statistically significant differences vs. modified membranes with PS NPs (after 1h ), p < 0.05.

Tab. S4. The zeta potential of *K. pneumoniae* bacterial cells after exposure to polystyrene nanoparticles PS NPs (20 µg/ml). Tests were carried out immediately after sample preparation (marked as control), and after 1 and 3 hours.

| pH | ***K. pneumoniae*** | | |
| --- | --- | --- | --- |
|  | control | 1h | 3h |
| 3.0 | -13.95 ± 1.20 | -11.85 ± 1.20^a^ | -13.05 ± 1.20 |
| 4.0 | -22.40 ± 1.20 | -18.78 ± 0.85^a^ | -21.18 ± 1.00^b^ |
| 5.0 | -28.10 ± 1.35 | -24.65 ± 1.89^a^ | -24.10 ± 0.95^a^ |
| 6.0 | -28.70 ± 1.25 | -27.35 ± 1.97 | -24.50 ± 0.98 |
| 7.0 | -29.00 ± 1.53 | -27.50 ± 1.20 | -23.90 ± 1.32^b^ |
| 8.0 | -29.30 ± 1.35 | -27.00 ± 0.85 | -25.00 ± 1.13^a,b^ |
| 9.0 | -30.05 ± 1.70 | -27.30 ± 0.75^a^ | -24.00 ± 1.20^a,b^ |
| 10.0 | -31.35 ± 1.06 | -27.50 ± 1.20^a^ | -23.95 ± 1.20^a,b^ |
| 11.0 | -31.87 ± 1.31 | -26.75 ± 1.10^a^ | -23.20 ± 0.90^a,b^ |

^a^ Statistically significant differences vs. control group, p < 0.05. ^b^ Statistically significant differences vs. modified membranes with PS NPs (after 1h ), p < 0.05.

Tab. S5. The zeta potential of *S. aureus* and *K. pneumoniae* bacterial cells after exposure to polystyrene nanoparticles PS NPs (20 µg/ml). Tests were carried out immediately after sample preparation (marked as control), and after 1 and 3 hours.

| **C_PS NP_= 0.4 [μg/ml]** | | | | |  |  |  |
| --- | --- | --- | --- | --- | --- | --- | --- |
| Bacterium | t [h] | *ζ*_pH = 5.0_ [mV] | *ζ*_pH= 7.4_ [mV] | *ζ*_pH= 9.0_ [mV] |  |  |  |
| *S. aureus* | 0.0 | -31.83 ± 1.47 | -28.33 ± 0.91 | -28.30 ± 1.16 |  |  |  |
|  | 0.5 | -29.85 ± 1.77 | -28.68 ± 1.33 | -27.50 ± 0.71 |  |  |  |
|  | 1.0 | -30.60 ± 1.09 | -27.43 ± 2.52 | -29.58 ± 1.89 |  |  |  |
|  | 3.0 | -26.05 ± 3.00^a,b,c^ | -26.05 ± 1.21 | -24.93 ± 1.14^a,c^ |  |  |  |
|  | 5.0 | -19.45 ± 0.64^a,b,c,d^ | -26.15 ± 2.25 | -28.55 ± 3.34^d^ |  |  |  |
| *K. pneumoniae* | 0.0 | -24.98 ± 1.10 | -25.83 ± 2.44 | -26.50 ± 0.65 |  |  |  |
|  | 0.5 | -24.93 ± 0.54 | -26.90 ± 1.15 | -27.18 ± 0.66 |  |  |  |
|  | 1.0 | -22.23 ± 1.37 | -26.90 ± 0.73 | -26.38 ± 1.27 |  |  |  |
|  | 3.0 | -25.35 ± 0.79^c^ | -24.78 ± 2.03 | -24.23 ± 0.91^b^ |  |  |  |
|  | 5.0 | -22.30 ± 1.12^d^ | -25.03 ± 1.59 | -24.88 ± 0.70 |  |  |  |
| **C_PS NP_ = 2 [μg/ml]** | | | | |  |  |  |
| Bacterium | t [h] | *ζ*_pH= 5.0_ [mV] | *ζ*_pH= 7.4_ [mV] | *ζ*_pH= 9.0_ [mV] |  |  |  |
| *S. aureus* | 0.0 | -42.83 ± 1.04 | -32.17 ± 0.65 | -27.95 ± 1.18 |  |  |  |
|  | 0.5 | -29.35 ± 1.01^a^ | -26.33 ± 0.83^a^ | -26.70 ± 1.36 |  |  |  |
|  | 1.0 | -22.33 ± 1.04^a,b^ | -21.80 ± 1.20^a,b^ | -21.80 ± 0.85^a,b^ |  |  |  |
|  | 3.0 | -18.75 ± 0.87^a,b,c^ | -20.30 ± 1.09^a,b^ | -22.35 ± 0.97^a,b^ |  |  |  |
|  | 5.0 | -17.78 ± 1.12^a,b,c^ | -22.93 ± 2.96^a,b^ | -26.60 ± 4.12^c,d^ |  |  |  |
| *K. pneumoniae* | 0.0 | -23.45 ± 1.37 | -24.70 ± 1.26 | -27.60 ± 1.19 |  |  |  |
|  | 0.5 | -26.33 ± 1.28 | -26.67 ± 0.68 | -25.03 ± 1.36 |  |  |  |
|  | 1.0 | -25.30 ± 1.36 | -27.17 ± 0.91 | -25.98 ± 0.61 |  |  |  |
|  | 3.0 | -23.60 ± 0.38^b^ | -22.57 ± 1.75^b,c^ | -24.25 ± 0.25^a^ |  |  |  |
|  | 5.0 | -23.03 ± 2.29^b^ | -20.17 ± 1.92^a,b,c^ | -23.23 ± 1.22^a^ |  |  |  |
| **C_PS NP_ = 20 [μg/ml]** | | | | |  |  |  |
| Bacterium | t [h] | *ζ*_pH = 5.0_ [mV] | *ζ*_pH = 7.4_ [mV] | *ζ*_pH = 9.0_ [mV] |  |  |  |
| *S. aureus* | 0.0 | -38.30 ± 0.53 | -34.60 ± 0.96 | -35.78 ± 0.43 |  |  |  |
|  | 0.5 | -38.48 ± 1.16 | -37.45 ± 0.41 | -32.50 ± 1.35^a^ |  |  |  |
|  | 1.0 | -30.05 ± 1.19^a,b^ | -33.25 ± 1.39^b^ | -36.68 ± 0.80^b^ |  |  |  |
|  | 3.0 | -20.33 ± 1.23^a,b,c^ | -26.65 ± 2.78^a,b,c^ | -27.80 ±3.09^a,b,c^ |  |  |  |
|  | 5.0 | -10.85 ± 1.57^a,b,c,d^ | -20.38 ± 1.79^a,b,c,d^ | -19.78 ± 2.67^a,b,c,d^ |  |  |  |
| *K. pneumoniae* | 0.0 | -23.93 ± 0.67 | -27.80 ± 0.76 | -29.38 ± 0.74 |  |  |  |
|  | 0.5 | -30.05 ± 0.55^a^ | -28.20 ± 1.15 | -27.55 ± 0.98 |  |  |  |
|  | 1.0 | -25.03 ± 2.63^b^ | -25.90 ± 0.39 | -26.13 ± 0.60^a^ |  |  |  |
|  | 3.0 | -19.78 ± 0.76^a,b,c^ | -20.60 ± 1.15^a,b,c^ | -19.15 ± 1.34^a,b,c^ |  |  |  |
|  | 5.0 | -18.40 ± 1.19^a,b,c^ | -20.58 ± 2.52^a,b,c^ | -19.25 ± 2.50^a,b,c^ |  |  |  |
| **C_PS NP_ = 100 [μg/ml]** | | | | |  |  |  |
| Bacterium | t [h] | *ζ*_pH = 5.0_ [mV] | *ζ*_pH = 7.4_ [mV] | *ζ*_pH = 9.0_ [mV] |  |  |  |
| *S. aureus* | 0.0 | -37.18 ± 0.96 | -35.40 ± 0.50 | -38.03 ± 1.68 |  |  |  |
|  | 0.5 | -34.10 ± 0.97^a^ | -41.65 ± 0.96^a^ | -44.98 ± 0.96^a^ |  |  |  |
|  | 1.0 | -38.28 ± 1.03^b^ | -41.58 ± 1.13^a^ | -44.93 ± 1.87^a^ |  |  |  |
|  | 3.0 | -33.13 ± 1.53^a,c^ | -39.63 ± 1.20^a^ | -40.35 ± 1.42^b,c^ |  |  |  |
|  | 5.0 | -34.55 ± 3.28^c^ | -40.48 ± 3.57^a^ | -45.90 ± 4.52^a,d^ |  |  |  |
| *K. pneumoniae* | 0.0 | -30.63 ± 0.96 | -32.03 ± 2.29 | -30.88 ± 0.84 |  |  |  |
|  | 0.5 | -26.25 ± 4.01^a^ | -34.40 ± 1.54 | -23.35 ± 2.31 |  |  |  |
|  | 1.0 | -32.95 ± 0.99^b^ | -42.05 ± 1.52^a,b^ | -43.10 ± 1.20^a,b^ |  |  |  |
|  | 3.0 | -37.33 ± 1.85^a,b,c^ | -40.53 ± 2.44^a,b^ | -43.90 ± 2.00^a,b^ |  |  |  |
|  | 5.0 | -35.85 ± 2.66^a,b,c^ | -42.23 ± 2.05^a,b^ | -41.88 ± 1.69^a,b^ |  |  |  |

^a^ Statistically significant differences vs. control group (t = 0.0 h), p < 0.05. ^b^ Statistically significant differences vs. modified membranes with PS NPs (t = 0.5 h ), p < 0.05. ^c^ Statistically significant differences vs. modified membranes with PS NPs (t = 1.0 h ), p < 0.05. ^d^ Statistically significant differences vs. modified membranes with PS NPs (t = 3.0 h), p < 0.05.

Table S6. Size of bacteria analyzed in this study.

| **Bacterium** | **Size [µm]** | |
| --- | --- | --- |
|  | **AFM** | **DLS** |
| *Staphylococcus aureus* strain ATCC 6538 | 1.00 – 1.83 (diameter)  1.25 – 1.92 (length) | 1,82 ± 0,40 (diameter) |
| *Klebsiella pneumoniae* strain  ATCC 4352 | 1.25 – 2.00 (diameter)  2.67 – 3.50 (length) | 3,61 ± 0,61 (diameter) |

Fig. S2. Size distribution by intensity of *S. aureus* strain ATCC 6538, *K. pneumoniae* strain ATCC 4352 and the polystyrene nanoparticles assessed using DLS in 0.3 mM NaCl.
